# A Systematic Review of Guidelines for Emergency Department Care of Sexual Minorities: Implementable Actions to Improve Care

**DOI:** 10.5811/westjem.20355

**Published:** 2025-03-13

**Authors:** Michael I. Kruse, Sawyer Karabelas-Pittman, Grace Northrop, Joanna Stuart, Suneel Upadhye, Blair L. Bigham

**Affiliations:** * McMaster University, Department of Family Medicine, Hamilton, Ontario, Canada; † Queen’s University School of Medicine, Faculty of Health Sciences, School of Medicine, Kingston, Ontario, Canada; ‡ University of Ottawa School of Medicine, Faculty of Medicine, Ottawa, Ontario, Canada; § University of British Columbia, Faculty of Medicine, Vancouver, British Columbia, Canada; ¶ University of Toronto, Dalla Lana School of Public Health, Toronto, Ontario, Canada; || McMaster University, Division of Emergency Medicine, Hamilton, Ontario, Canada; # Scarbrough Health Network, Department of Critical Care. Toronto, Ontario, Canada

## Abstract

**Introduction:**

Sexual minorities, including lesbian, gay, bisexual, asexual, pansexual, and others make up 4.0–5.4% of the North American population. Stigmatization and minority stress can lead to poorer health status in sexual minorities, and a previous scoping review showed gaps in the emergency medicine (EM) literature for care of sexual minorities. In this review we sought to examine existing guidelines for the care of sexual minorities that made recommendations relevant to care in the emergency department (ED).

**Methods:**

Using the PRISMA criteria, we performed a systematic search of OVID Medline, EMBASE, CINAHL, and the grey literature for clinical practice guidelines (CPG) and best practice statements (BPS) published until July 31, 2022. Articles were included if they were in English, included medical care of sexual minority populations of any age, in any setting, region, or nation, and were of national or international scope. Exclusion criteria included primary research studies, systematic or narrative reviews or otherwise non-CPG or BPS statements, editorials or letters to the editor, articles of regional or individual hospital scope, non-medical articles, or if a more recent version of the CPG or BPS existed. We identified, recorded, and assessed for quality all recommendations relevant to using the AGREE-II and AGREE-REX tools. Inter-rater reliability was assessed using the interclass correlation coefficient. We coded recommendations for the relevant point of care while in the ED (triage, registration, rooming, investigations, etc).

**Results:**

We excluded 2,413 of 2,534 unique articles. Only nine articles contributed 11 ED-relevant recommendations. Seven of the nine articles were found to be of moderate to high quality; 6 of 11 recommendations were identified as high quality and adaptable. They included recommendations for screening, testing, and care of HIV+ sexual minority populations, and general or trauma care for men who have sex with men and sexual minority populations in general.

**Conclusion:**

This is the most comprehensive review of guidance documents for care of sexual minority populations to date. It identifies 11 actionable recommendations for the ED and identifies opportunities for community-led development of comprehensive clinical practice guidelines for care of sexual minority populations in the ED.

## INTRODUCTION

Gay, lesbian, bisexual, and other identities that comprise sexual minorities^1^ represent between 4.0–7.6% of the North American population, with the majority identifying as bisexual.^2^,^3^ This has been increasing over time, and led by “Generation Z,” born between 1997–2002, 16% of whom report a non-heterosexual identity.^3^

In jurisdictions that lack universal health care and/or have restrictive marriage laws, a sexual minority person will have reduced access to health insurance.^4^ Sexual minorities may experience social marginalization, and there are fewer culturally competent healthcare clinicians to foster safe environments for care for sexual minorities. As a result, there is still a reluctance to disclose this identity to clinicians.^4^–^6^ Further, sexual minorities have poorer overall health compared to their heterosexual counterparts as a result of prejudice, marginalization, and minority stress,^7^–^9^ contributing to increased risk for suicidal ideation^10^,^11^ and substance use disorders,^13^,^14^ and more risk factors for cardiovascular disease.^15^ They are also at increased risk for breast and anal cancer in lesbians and gay men, respectively, as a result of decreased screening and environmental and lifestyle differences, compared to heterosexual populations.^12^ These barriers to healthcare and greater disease risks mean that sexual minorities may rely more heavily on the emergency department (ED) for their primary care, and/or may delay seeking care until their health becomes poor enough that they need emergency care.^4^ It is imperative that these barriers are not reinforced in the ED.

Clinical practice guidelines (CPG) are collaborative, structured guidance documents described by the US Institute of Medicine as “statements that include recommendations intended to optimize patient care that are informed by a systematic review of evidence and an assessment of the benefits and harms of alternative care options.”^16^ Best practice statements (BPS), which are more difficult to define, can include a practice advisory or consensus statement from professional societies or leaders in front-line care.^17^ Both documents represent standardized approaches to evidence-based care and are often adapted to meet the more focused needs of front-line clinicians in the form of clinical manuals. While there have been reviews of guidelines for care of sexual minorities, none focus on ED-relevant recommendations.^18^,^19^

Previous work has shown there is a limited amount of research relevant to the care of sexual minorities in the ED.^20^ This article is the second of three systematic reviews and quality assessments of guidelines for the care of sexual and gender minorities in the ED and focuses on ED-relevant recommendations for care of sexual minorities. The first review^21^ focused on transgender populations, and the third will focus on intersex populations.

## METHODS

This study followed the PRISMA guidelines for systematic reviews, using the AGREE-II (Appraisal of Guidelines for Research and Evaluation-II) and AGREE-REX (Recommendations Excellence) tools for evaluation of quality and clinical applicability (available at www.agreetrust.org). This trial was registered at the Open Science Foundation before commencement and can be found at https://doi.org/10.17605/OSF.IO/ZPMEK. A comprehensive search of Medline, EMBASE, and CINAHL, performed in collaboration with a medical librarian, included any article published up until July 31, 2022, using keywords relating to the sexual minority population and guidelines (Appendix C). We combined a grey literature search using Google Scholar with a search of relevant societies and clinical groups for clinically focused statements and guidelines to support the automated search.

Articles were included if they were a CPG, BPS (Box A), consensus document, or other formalized guidance for clinical care of sexual minorities of any age (eg, lesbian, gay, bisexual, asexual, or pansexual populations), in English, in any practice setting, and were of regional, national, or international scope. Articles were excluded if they were replaced by a later version of the guideline, were a systematic or narrative review, offered unstructured or non-medical guidance, or if they were local, municipal, or single institution in scope.

Box A **Key features of a clinical practice guideline and best practice statement.^22^–^24^**

**Clinical Practice Guidelines** are statements that include recommendations intended to optimize patient care that are informed by a systematic review of evidence and an assessment of the benefits and harms of alternative care options and contain the following features:

Essential Features:

- Broad stakeholder involvement of all relevant parties
- Explicit conflict of interest statements presented
- Clear questions to specifically guide clinical practic
- Thorough, transparent retrieval and assessment of evidence; may have an accompanying systematic review/meta-analysis to inform recommendations
- Structured grading of evidence and framing of recommendations using accepted framework (eg, GRADE)
- External review by relevant bodies
- Key recommendations highlighted in document
- Updating timelines presented
- Reporting using AGREE-II framework

Desirable Features:

- Implementation protocols/pathways provided for end-users
- Outcome measurement tools provided; audit and feedback processes recommended

**Best Practice Statements** are consensus statements, practice advisories, position statements, position papers, or frontline clinical manuals usually from professional societies or specialist groups that have the following features:

- Current important topic for practice
- Attempt to seek and evaluate evidence
- Practical recommendations to guide practice
- High level of certainty that recommendations will improve patient care

*AGREE-II*, Appraisal of Guidelines for Research and Evaluation II; *GRADE*, Grading of Recommendations Assessment, Development, and Evaluation.

Two of four reviewers independently screened every title/abstract and then included full-text articles (MK, SKP, GN, JS) using Covidence (covidence.org). Conflicts were resolved by a group consensus meeting, with ties broken by senior reviewer MK. Included studies were abstracted and analyzed for ED-relevant recommendations using a keyword search for the term “emergency.” The ED-relevant recommendations were coded as a CPG or BPS, by their country or region of origin, and by their domain(s) of relevance to the ED: decision to come to ED; prehospital care; registration; triage; waiting room experience; rooming/initial nursing care; history and physical exam; investigations; diagnoses; treatment; and disposition/discharge planning and/or follow-up care. This was done by two of four independent researchers (MK, SKP, GN, JS), and conflicts were resolved by a group consensus meeting, with ties broken by senior reviewer MK.

The methodological quality of the guidelines included was assessed using the AGREE-II instrument (four reviewers: MK, SKP, GN, JS), and of the included recommendations using the AGREE-REX instrument. Raters received training in the use of the instruments through both an online tutorial available at McMaster University,^25^ and from senior researchers on the project. We calculated rating scores using the online AGREE-II calculator (downloaded for free from the AGREE Trust website above) and with Microsoft Excel (Microsoft Corp, Redmond, WA) for the AGREE-REX instrument. Comparators were by individual raters, rather than by consensus, and were reported in the percent total score of each domain (Appendix A, Figures C and D). More specifically, the score is calculated by first summing all the appraisers’ scores in one item or sub-domain, then adding all the summed sub-domain scores together and scaling that number as a percentage of the maximum possible score in the domain (Appendix A, Figure 2). As per the AGREE-II and AGREE-REX instruments,^26^,^27^ we gave overall scores of >70% a high-quality rating, a score of ≥ 30 and ≤ 70% a moderate-quality rating, and a score of < 30% a low-quality rating. We assessed inter-rater reliability using the intraclass correlation coefficient (ICC) statistic in SPSS Statistics for Windows v28 (IBM Corporation, Armonk, NY). An ICC score of <0.50 was considered poor, from 0.50 to <0.75 moderate, from 0.75 to <0.9 good, and > 0.90 excellent.^28^

## RESULTS

**Figure 1:**
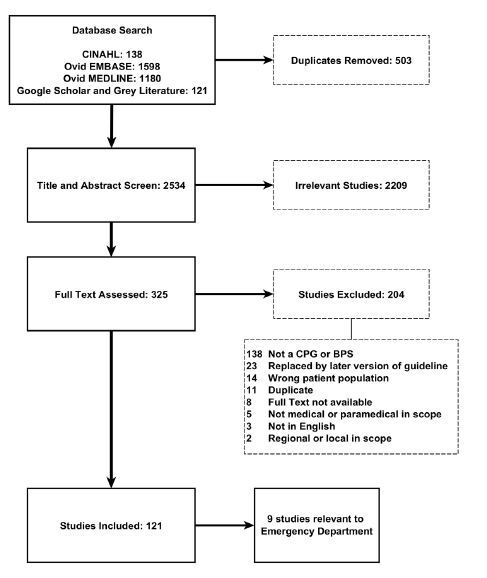
PRISM diagram. *CPG*, clinical practice guideline; *BPS*, best practice statement; *PRISM*, Practical, Robust Implementation and Sustainability Model.

Our search yielded a total of 3,037 studies. After we removed 503 duplicates, we screened the titles and abstracts of 2,534 studies and included 325 for full text review. Of these, we excluded 204 studies, and 121 studies underwent keyword search and abstraction (Figure 1, see Appendix B for complete list). Nine^29^–^37^ of the included studies (Appendix A Table 1) yielded the 11 ED-related recommendations (Box B), and these were evaluated for quality and clinical applicability using the AGREE-II and AGREE-REX instruments, respectively.

Box B **Summary of recommendations.^29^–^37^**

**HIV Care**

1. Emergency clinicians should use rapid testing technology to ensure STI diagnoses and ensure that post-test counselling reaches men who have sex with men (MSM) clients. (Ad Hoc Expert Working Group for CDC 2012).
2. Education for ED staff and local protocols are required to ensure appropriate advice and baseline HIV testing for MSM requesting post-exposure prophylaxis (PEP) following HIV exposure. (Clutterbuck et al 2018).
3. MSM should be routinely offered testing for HIV in the ED in areas of high prevalence whether they are undergoing venipuncture for another indication or not (Palfreeman et al 2020).
4. Medications for non-occupational PEP should be readily available in the ED if they are needed urgently (Tan et al 2017).

**Visitation Policies**

5. All medical facilities should allow patients to determine who may visit and act on their behalf, regardless of sexual orientation (Daniel et al 2015).

**Prehospital Care**

6. Reduce the proportion of LGB persons who delay or have difficulty in getting emergency medical care. (GLMA 2010).
7. Increase the proportion of LGB persons who have access to prehospital emergency services (GLMA 2010).
8. Increase the districts with trauma systems that maximize, prevention, survival, and functional outcomes of LGB trauma patients (GLMA 2010).

**Interpersonal Violence**

9. Medical centres and clinicians ought to be prepared to help LGB people find support when they are survivors of intimate partner violence. (National LGBTQIA+ Health Education Centre 2019).

**Not Otherwise Categorized**

10. Create a culturally sensitive medical home and referral pathway for sexual minority people who use the ED for primary care (Bell et al 2021).
11. During sexual counselling, LGB patients should be advised to seek emergency care should they experience angina during sex that does not resolve spontaneously in 15 minutes or 5 mins after nitrate use and call 9-1-1 should they not be able to use nitrates. (Steinke et al 2013).

*Gay and Lesbian Medical Association (GLMA)*, Health Professionals Advancing LGBTQ+ Equality; *LGB*, lesbian, gay, bisexual; STI, sexually transmitted infection.

Five of the nine articles were classified as BPS,^29^,^31^,^34^,^36^,^37^ and four as CPG.^30^,^32^,^33^,^35^ Four of the articles concerned screening, diagnosis, and care of HIV+ populations,^30^,^32^,^36^ while two covered general medical and/or trauma care for sexual and gender minority populations.^34^,^37^ One BPS examined the health inequalities of American Indian and Alaska Native [*sic*] children and included sexual minority populations as a subgroup,^29^ as did a guideline on sexual counselling for people with cardiovascular disease and their partners.^35^ Finally, there was a CPG on caring for the sexual health in general of men who have sex with men^34^ and a UK guideline on intimate partner violence (IPV) in sexual and gender minority populations.^32^ Domains of care in the ED covered by these recommendations include the decision to come to the ED, triage and rooming, investigations and treatments, and disposition and follow-up care, with the majority focusing on investigations and treatments.

The overall quality of the documents and recommendations are to be found in Appendix A and Box B in Appendix A. Most guidance documents scored moderate or high in quality rating, and two BPS^29^,^31^ scored low, due to poor rigor of development and applicability. The AGREE-REX scoring found that all the recommendations were of moderate to high quality, but only nine of the 12 recommendations could be adopted (1–6, 9, 11), while the remaining three (7, 8, 10) could not find a consensus. Inter-rater reliability using interclass correlation coefficient (ICC) for the AGREE-II ratings (Appendix, Tables 3 and 4) showed good or excellent correlation between raters for most domains, with only scope and purpose showing poor correlation. The ICC for the AGREE-REX domains were good to moderate for clinical applicability, values, and preferences, and the total score, but implementability had poor correlation.

## DISCUSSION

This is the largest and most comprehensive review of guidance for care of sexual minorities in the ED to date, identifying 122 international CPG or BPS, while prior reviews found only 11–17.^18^,^19^ We found 11 moderate-to-high quality recommendations that could be implemented now in most EDs.

### HIV Care

Recommendations for care of sexually transmitted infection/HIV+ and at-risk sexual minorities are the focus of over one third of the recommendations (1–4). The HIV crisis continues among sexual minority communities, with MSM at greatest risk.^38^,^39^ In the US, it was estimated that in 2023, 156,000 HIV+ people were not diagnosed or engaged in care.^40^ In Canada, this number is estimated to be 16,969^39^ with a large percentage of those in the US (67%) and Canadian (50.3%) populations being MSM. 39–40 Black and Latinx MSM are at particular risk in the US and have an increased disease burden and a decreased opportunity of being tested or diagnosed.^41^ This represents an opportunity to identify new HIV+ MSM and connect them to services to extend life, reduce long-term healthcare costs, and prevent the spread of HIV.

It is recommended to target MSM populations for HIV screening in high prevalence areas. However, non-targeted screening, also called universal screening, for all sexually active people ages 13–65 is currently recommended by multiple North American agencies.^42^–^44^ This allows detection before symptoms develop, with infected people gaining years of life and showing economic benefit.^45^ The recommendations in our review focus on MSM populations and suggest that they may need to be specifically targeted for screening, treatment and referral. However, risk-based assessment may increase stigma and is dependent on patients self-reporting risk, the likelihood of which is dependent on the trust of the local system.^46^–^47^

The guidance for universal screening does not offer a standard approach to the development of HIV screening programs.^46^ There have been both experimental and government-mandated ED-based HIV testing programs in large urban areas in Canada, the United Kingdom (UK), and the US that show such programs can increase the testing yield and be cost effective,^48^ especially if they are opt-out programs.^45^ One facilitator of HIV testing in the ED would be access to rapid testing, as per Recommendation 1. In a New York-based study, adolescent patients were much more likely to seek HIV testing in the ED if there was a rapid test available,^49^ and a recent commentary that outlined the barriers and facilitators of point-of-care rapid testing in the ED declared it a reasonable and realistic goal.^50^

There are alternatives to universal or targeted screening to HIV in the ED. Anonymous testing for HIV is available in many jurisdictions, but its successes are mixed and dependent on local factors.^51^ This would also require new ED information technology infrastructure and creative solutions to patient communication that maintain privacy. Alternatively, effective HIV self-testing kits are currently available in the US and Canada.^52^ While home-based self-testing for those who do not want to disclose their MSM status in the ED can help, it raises the risk of losing direct linkage to support and treatment because it would be up to the individual to self-refer and they may need more support and counselling to do so. 54,55 Finally, testing for HIV when people present with indicator conditions that are more associated with HIV infection, such as syphilis and gonorrhea, may increase the testing uptake.^53^ Both universal HIV screening and these alternatives require creative solutions to linkage to care, and it is not clear whether one is superior to the other.

Improving access to non-occupational post-exposure prophylaxis (nPEP) is needed; it is under-prescribed in the ED. Barriers to prescribing nPEP from the ED include lack of clinician time for assessment and counselling, difficulty in connecting patients to follow-up, and cost of the medication for the patient.^56^ The implementation of nPEP programs is not standardized, with different recommendations for timing of initiation, regimens, and referral for nPEP adherence and completion.^57^ Despite this, nPEP programs do exist in several jurisdictions, usually in the context of sexual assault.^58^ A national standard for nPEP with robust referral pathways is sorely needed, along with education to increase awareness of nPEP among patients and clinicians.

Initiation of pre-exposure prophylaxis (PrEP) in the ED is also recommended. Several barriers to eligibility screening, such as lack of clinician training, lack of effective and equitable screening tools to identify higher risk individuals, and proven clinical models for provision, lead to uneven distribution, with one study showing 67% of new prescriptions in Ontario from 2015–2018 were filled in Toronto.^59^,^60^ There is a similar trend in the US, with the southeastern states responsible for over one half of new HIV diagnoses but only one quarter of PrEP-providing clinics.^61^ Black and Latinx populations are also underserved.^62^ Universal HIV screening offers an opportunity to offer referral to PrEP clinics for at-risk people, and a recent review showed that rapid or same-day referral to PrEP clinics increased uptake.^63^

The implementation of rapid HIV testing would also facilitate PrEP prescribing, as a reliable rapid test would allow the initiation of PrEP in HIV-individuals directly from the ED. As of yet, reviews of the economic feasibility have focused on the HIV positivity rate but have not factored in the benefits of starting PrEP earlier, thereby avoiding HIV infection altogether.^47^,^64^ Future studies should include the benefits of prevention when studying rapid testing, which may tip the balance to offering this as a standard in all EDs and support PrEP programs in the ED in both the US and Canada.

With the growing crises of lack of access to primary care,^65^,^66^ it is important to build robust pathways to referrals for sexual minorities who test positive for HIV in the ED or present to the ED HIV+ and undertreated. The continuity of improved ED-based care is attenuated if appropriate post-ED follow-up care is not available after discharge. A 2016 review found 37 linkage-to-care (LTC) programs in the US and included various strategies, such as physically escorting the patient to HIV clinics and referrals to outside clinics, with the former demonstrating improved LTC with a rate greater than 85%, compared to the average of 74%.^67^ One of the limitations of LTC programs is that they require a lot of infrastructure and multidisciplinary teams and are often integrated into larger HIV screening, counselling, rapid treatment, and referral programs that need systematic changes to be successful.^53^ To implement this widely there would need to be a systematic approach at the state, provincial, or national level to develop, fund, implement and monitor the success of such programs. Given the large number of undiagnosed HIV+ people in the US and Canada, this is a necessary next step.

In sum, the HIV recommendations speak to the need in every ED for a robust, well-supported HIV testing program that offers universal rapid testing in the ED while at the same time increasing the safety for disclosure of sexual minority status. This will decrease stigma, increase uptake of pre- and post-exposure HIV prophylaxis, and improve connections to treatment clinics and counselling to support new or undertreated HIV diagnoses.

### Visitation Policies

Recommendation 5 identifies different family structures that sexual minorities develop to build community and share their lives and that will be involved in their healthcare. While same-gender marriages are recognized in all jurisdictions in the US and Canada, some sexual minority people may not pursue marriage and, as they age, they may be relying on informal care networks like those established during the AIDS crises in the 1980s and 90s.^68^,^69^ Equal visitation polices are mandated for facilities funded by the US Centers for Medicare & Medicaid, and 99% of hospitals participating in the Healthcare Equity Index survey document such policies.^70^,^71^ Visitation policies that reflect all types of families and caregivers should be implemented to support sexual minorities presenting to the ED.

### Prehospital Care

Recommendations 6–8 focus on prehospital care: first responders are the bridge between community and hospital care. Since the publication of the Health People Companion Document for LGBT Health in 2010, recommendations for sexual and gender minorities have been added to the main Health People document, but they do not include any statements about prehospital care.^37^,^72^ There is no evidence that prehospital barriers to care for sexual minorities have lessened since 2010; thus, when developing systems to remove barriers in the ED, emergency medical services should be included in these systems.

### Interpersonal Violence

Recommendation 9 addresses the bias sexual minorities face when seeking care for IPV and others forms of violence. Emergency departments need to include screening for IPV in all demographics when patient present to the ED so that we have a greater chance of identifying these invisible populations. Gay men are subject to higher rates of IPV than heterosexual men and women, with rates as high as 26–33%. The risk was higher if they were also a person of color, HIV+, and were younger. About 13–40% of lesbians and bisexual women report physical violence, and 11–14% report sexual violence within same-sex relationships.^73^ It is important to recognize that the stigma that sexual minority populations face along with barriers to care may decrease reporting of IPV in the ED. Harland et al reported a less than expected reporting of IPV among sexual minorities in the ED than heterosexual counterparts This may have been due to the lack of access to care, fear of disclosure of sexual minority relationships, discrimination upon disclosure, and a focus in screening for IPV in heterosexual relationships.^74^

As well, referrals for care when IPV is identified need to be appropriate for the sexual minority and may differ from heterosexual populations. There are significant barriers in seeking shelter for gay, lesbian, and bisexual people in a same-gender relationship. A 2016^75^ review of barriers to care in US sexual and gender minority (SOGI) populations found that 61.6% of SOGI IPV survivors were turned away from shelters when seeking assistance, and none of the 1,500 domestic violence (DV) shelters in the US are dedicated to lesbian IPV survivors. Among the reasons lesbian IPV survivors gave regarding their fear of seeking help in DV shelters was that their abusive partners would be able to find them and seek access at the same shelters. This is an opportunity for EDs to work with IPV referral partners to make sexual minority training mandatory to ensure a safe and appropriate space for sexual minority IPV survivors to seek shelter.

### Not Otherwise Categorized

Referrals to primary care are a challenge from the ED, given the limitations of access as described previously in this paper. Building relationships with culturally humble clinicians in the community and with the community itself can build trust and remove barriers for care in the ED, but this remains an aspirational goal with all the other pressures on referrals from the ED. As to the recommendation for angina counselling, this can be considered when discharging a sexual minority person at risk for cardiovascular disease, although it is dependent upon creating an environment that is felt permissible for disclosure of sexual minority status. Also, this is good advice for any person, sexual minority or not, who is at risk for cardiovascular disease.

## LIMITATIONS

This systematic review of guidelines is limited by exclusion of recommendations not directly related to care in the ED; there may be guidelines and recommendations that are focused on other aspects of community or hospital care that can inform care in the ED. Indeed, the included studies were focused on primary or specialist care, and not the ED, and we did not analyze the indirect applicability of the other guidelines in the documents. Counter to this, because the recommendations were not developed on primary ED populations they may not be applicable in the ED. This is the focus of future work in the development of ED guidelines for the care of sexual minorities. Only English articles were included. Despite a grey literature search, we may have missed relevant BPS that are typically not part of searchable databases. Finally, the age of some of the recommendations may preclude their use in the current ED environment as social and legislative changes may result in different approaches if the same recommendations were to be developed today.

The AGREE-REX findings may be less reliable because we used four independent raters, while the tool was validated using five. Also, while the AGREE-II and AGREE-REX tools do include a risk of bias assessment (see section 9)^26^ they do not capture this more subtle source of bias in guideline development.^76^

While the recommendations touched on most elements of the domains of care, they do not represent a comprehensive pathway of experience through an ED visit and leave out crucial experiences that may reinforce barriers to care, including sexual orientation screening at registration and training of front-line staff other than clinical staff. This also highlights a more general limitation of the lack of ED-focused recommendations in general. As identified in our previous scoping review there are still many gaps in the literature pertaining to care of sexual minority populations in the ED.^20^ The recommendations this review identified do not speak to the collection of gender, sex, or sexual identity demographics at registration or triage, identify any disease-specific recommendations other than HIV, and they did not include sexual minority populations as stakeholders in the design and implementation of any of the guidance documents.

Inter-rater reliability (ICC) was poor for the implementability of these recommendations, according to the AGREE-REX evaluation. This is most likely due to the varied stages of medical training for the evaluators, which included medical students, residents, and staff physicians. It is likely that due to the different experience levels, there was uncertainty about whether these recommendations could be implemented in the clinical context of the individual rater. With raters of more closely matched clinical experience, we would expect the ICC to be better.

### Next Steps

These 11 recommendations are actionable. They can serve as a basis for development of CPG for care of sexual minorities in the ED and spark reflection and innovation. Our review highlights important gaps in the literature that may inform a future research agenda for care of sexual minorities in the ED.

An essential step in the adoption and implementation of these recommendations is the involvement of the sexual minority community in the development of any research questions, clinical trials, and guideline development. While there were advisory boards of community members participating in some of the guideline development process, they have not participated in the development of the research questions and priority setting for the guideline development group. In fact, due to past and ongoing discrimination of sexual minorities in medicine in general,^77^ the disconnect between the community and the evidence base for care is built into the knowledge creation system.^20^ We have engaged sexual minority members of the medical community in the production of this manuscript, but to re-design the entire guideline development process to be more equitable, our group is developing a Delphi-type^78^ process to engage the community in the identification of research priorities and study design. Our hope is this will broaden our CPG to serve the community it focuses on, rather than just the researchers or system it works within.

## CONCLUSION

This is the most comprehensive review of clinical practice guidelines and best practice statements for sexual minority populations to date and identifies 11 actionable recommendations for care of sexual minorities in the ED. It also reveals an opportunity for community-led implementation of these recommendations in an equitable manner to ensure that HIV testing, treatment, and referral pathways exist and do not reinforce barriers to care. There is a clear need for focused EM-relevant practice guidance for sexual minority patients in ED settings, ideally co-created with emergency clinicians and sexual minority community members.

## Supplementary Information
