# Supplementary material for: A Systematic Review of Guidelines for Emergency Department Care of Sexual Minorities: Implementable Actions to Improve Care
Source: West J Emerg Med. 2025 Mar 13;26(3):431–40. doi: 10.5811/westjem.20355 (PMC12208086; doi:10.5811/westjem.20355)
Supplement: Supplementary file 1 [file wjem-26-431-s001.docx]

# Appendix A

**Box C AGREE-II Domains***

**Domain 1. Scope and Purpose** is concerned with the overall aim of the guideline, the specific health questions, and the target population (items 1-3).

**Domain 2. Stakeholder Involvement** focuses on the extent to which the guideline was developed by the appropriate stakeholders and represents the views of its intended users (items 4-6).

**Domain 3. Rigour of Development** relates to the process used to gather and synthesize the evidence, the methods to formulate the recommendations, and to update them (items 7-14).

**Domain 4. Clarity of Presentation** deals with the language, structure, and format of the guideline (items 15-17).

**Domain 5. Applicability** pertains to the likely barriers and facilitators to implementation, strategies to improve uptake, and resource implications of applying the guideline (items 18-21).

**Domain 6. Editorial Independence** is concerned with the formulation of recommendations not being unduly biased with competing interests (items 22-23).

**Overall assessment** includes the rating of the overall quality of the guideline and whether the guideline would be recommended for use in practice.

***Reproduced directly from AGREE-II Manual, page 7. AGREE Next Steps Consortium. The AGREE II Instrument [Electronic version]. 2017 Accessed October 3, 2021. https://www.agreetrust.org/wp-content/uploads/2013/06/AGREE_II_Users_Manual_and_23-item_Instrument_ENGLISH.pdf**

**Table 1: AGREE-II Evaluation**

| **Author and Year** | **Title** | **Country or Region** | **CPG or BPS** | **Dom. 1 (%) Scope** | **Dom. 2 (%) Stake.** | **Dom. 3 (%) Rigor** | **Dom. 4 (%) Clarity** | **Dom. 5 (%) Applic.** | **Dom. 6 (%) Editor.** | **Overall Score** | **Overall Quality** |
| --- | --- | --- | --- | --- | --- | --- | --- | --- | --- | --- | --- |
| Ad Hoc Expert Working Group Centre for Communicable Diseases and Infection Control 2012 | Human Immunodeficiency Virus HIV Screening and Testing Guide | Canada | BPS | 86 | 69 | 19 | 82 | 47 | 19 | 58 | Moderate |
| Bell et al 2021 | Caring for American Indian and Alaska Native Children and Adolescents. | USA | BPS | 81 | 43 | 11 | 50 | 17 | 90 | 25 | Low |
| Clutterbuck et al 2018 | 2016 United Kingdom national guideline on the sexual health care of men who have sex with men | United Kingdom | CPG | 88 | 90 | 87 | 93 | 65 | 98 | 92 | High |
| Daniel et al 2015 | Lesbian, Gay, Bisexual, and Transgender Health Disparities: Executive Summary of a Policy Position Paper From the American College of Physicians | USA | BPS | 71 | 28 | 16 | 60 | 27 | 31 | 33 | Moderate |
| Gay and Lesbian Medical Association 2010 | Healthy People 2010: Companion Document for LGBT Health | USA | BPS | 83 | 74 | 19 | 81 | 52 | 44 | 54 | Moderate |
| National LGBTQIA+ Health Education Center 2019 | Recognizing and Addressing Intimate Partner Violence in Relationships of LGBTQ People: A Primer for Health Centers | USA | BPS | 60 | 22 | 6 | 63 | 22 | 33 | 25 | Low |
| Palfreeman et al 2020 | British HIV Association/British Association for Sexual Health and HIV/British Infection Association adult HIV testing guidelines 2020 | United Kingdom | CPG | 89 | 82 | 91 | 96 | 71 | 58 | 92 | High |
| Steinke et al 2013 | Sexual counselling for individuals with cardiovascular disease and their partners: a consensus document from the American Heart Association and the ESC Council on Cardiovascular Nursing and Allied Professions (CCNAP) | USA and Europe | CPG | 81 | 49 | 49 | 86 | 49 | 67 | 67 | Moderate |
| Tan et al 2017 | Canadian guideline on HIV pre-exposure prophylaxis and nonoccupational postexposure prophylaxis | Canada | CPG | 86 | 82 | 83 | 97 | 72 | 69 | 79 | High |

**Box D: The AGREE-REX Domains***

**Domain 1: Clinical Applicability**: the degree to which the recommendation is clinically supportable and applicable:

**Sub-domain 1: Evidence:** the degree to which the recommendation was based on available evidence.

**Sub-domain 2: Applicability to target users**: the degree to which the users of the recommendation can apply it.

**Sub-domain 3: Applicability to Patients/Populations:** the degree to which the recommendation is applicable to the target population.

**Domain 2: Values and Preferences:** the degree to which the recommendation incorporated the values and preferences of:

**Sub-domain 1: target users** of the recommendation.

**Sub-domain 2: target population** of the recommendation.

**Sub-domain 3: target policy/decision makers** of the recommendation.

**Sub-domain 4: guideline developers** of the recommendation.

**Domain 3: Implementability**: the degree to which the recommendation is able to be implemented in the target environment, by the target user, or in the specific target population:

**Sub-domain 1: Purpose:** the degree to which the recommendation aligns with the purpose of the guideline in which it is contained.

**Sub-domain 2: Local Application and Adoption:** the degree to which the recommendation can be applied and adopted in the setting/location or health system in which they are implemented.

**Adapted from the AGREE-REX instrument:** AGREE-REX Research Team. The Appraisal of Guidelines Research & Evaluation—Recommendation EXcellence (AGREE-REX) [Electronic version]. Accessed October 3, 2021. https://www.agreetrust.org/wp-content/uploads/2021/07/AGREE-REX-Tool-PDF-version.pdf

**Table 2: Individual Recommendations and AGREE-REX Ratings**

| Author Year | Recommendation | ED Visit Event | Domain 1 (%)  Clinical Applicability | Domain 2 (%)  Values and  Preferences | Domain 3 (%)  Implementability | Total (%) | Would you Use Recommendation?  (Yes/Yes with reservations/No) |
| --- | --- | --- | --- | --- | --- | --- | --- |
| Ad Hoc Expert Working Group Centre for Communicable Diseases and Infection Control 2012 | It is recommended that care providers operating in an emergency room setting take advantage of rapid testing technology to ensure STI diagnosis and post-test counselling reaches their clients. (MSM were identified as high priority group). | Investigations | 57 | 46 | 85 | 58 | Yes |
| Bell et al 2021 | Create a medical home sensitive to discrimination against Indigenous Sexual minorities. Work with ED’s to create referral pathway from ED for those who use ED for primary care. | Disposition, Follow-up Care | 40 | 19 | 63 | 36 | Yes, with reservations |
| Clutterbuck et al 2018 | Education of emergency department staff and local protocols are required to ensure appropriate advice, baseline HIV testing and follow-up for MSM who present requesting PEP following sexual exposure to HIV (PEPSE) | Investigations, Treatment, Follow-up Care | 89 | 57 | 77 | 72 | Yes |
| Daniel et al 2015 | All hospitals and medical facilities should allow patients to determine who may visit and act on their behalf, regardless of sexual orientation, or marital status, and ensure visitation policies are consistent with CMMS Conditions of Participation and JC standards for Medicare funded hospitals | Rooming/Initial Nursing Care | 43 | 23 | 63 | 38 | No consensus |
| Gay and Lesbian Medical Association 2010 | Reduce the proportion of LGB persons who delay or have difficulty in getting emergency medical care. | Decision to Come to ED | 39 | 42 | 56 | 44 | No consensus |
|  | Increase the proportion of LGB persons who have access to rapidly responding prehospital emergency medical services. | Prehospital Care | 44 | 38 | 52 | 43 | Yes, with reservations |
|  | Increase the number of tribes, States, and the District of Columbia with trauma care systems that maximize survival and functional outcomes of LGB trauma patients and help prevent injuries from occurring. | Triage AND Treatment | 39 | 39 | 48 | 41 | No consensus |
| National LGBTQIA+ Health Education Center 2019 | LGB survivors of intimate partner violence (IPV) often have barriers to access support services, health centers and individual providers ought to be prepared to help LGB people find supports for IPV | Rooming/Initial Nursing Care | 48 | 31 | 50 | 41 | Yes, with reservations |
| Palfreeman et al 2020 | MSM are at increased risk of exposure to HIV and should routinely offered testing, and if they are in areas of high or extremely high prevalence should be offered routine testing, whether or not they are undergoing venipuncture of another indication | Investigations | 96 | 70 | 88 | 82 | Yes |
| Steinke et al 2013 | During sexual counselling, LGB pts (as all patients) should be told to seek emergency care should they experience coital angina that does not resolve spontaneously in 15 mins, or 5 mins after nitrate use. If they are using PDE5 inhibitors hey should be counselled to avoid nitrates and call emergency medical services immediately if experience coital angina | Decision to come to ED | 71 | 21 | 71 | 49 | Yes |
| Tan et al 2017 | Medications for nPEP should be readily available in emergency departments where they are likely to be needed urgently. | Treatment | 57 | 46 | 85 | 58 | Yes |

**Figure 2: Sample Calculation from AGREE-II and AGREE-REX Instrument***

**
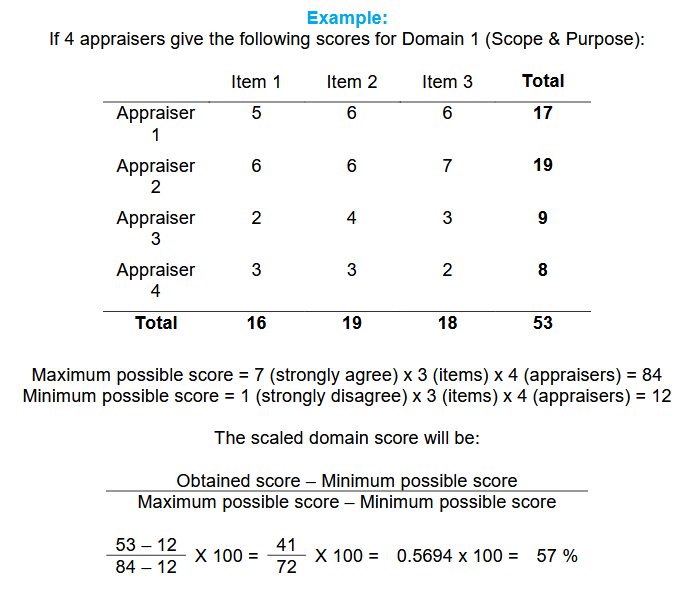
**

**From the AGREE-II instrument, page 13, AGREE Next Steps Consortium. The AGREE II Instrument [Electronic version]. 2017. Accessed October 3, 2021. https://www.agreetrust.org/wp-content/uploads/2013/06/AGREE_II_Users_Manual_and_23-item_Instrument_ENGLISH.pdf**

**Table 3: Interclass Correlation Coefficient by Domain AGREE-II**

| Agree II Domain | Interclass Correlation Coefficient | 95% Confidence Interval | Rating |
| --- | --- | --- | --- |
| 1: Scope and purpose | 0.29 | -0.97 to 0.82 | Poor |
| 2: Stakeholder involvement | 0.88 | 0.66 to 0.97 | Good |
| 3: Rigor of development | 0.97 | 0.91 to 0.99 | Excellent |
| 4: Clarity of presentation | 0.85 | 0.59 to 0.96 | Good |
| 5: Applicability | 0.75 | 0.29 to 0.94 | Good |
| 6: Editorial independence | 0.78 | 0.39 to 0.94 | Good |
| Totals | 0.95 | 0.85 to 0.99 | Excellent |

**Table 4: Interclass Correlation coefficient by Domain, AGREE-REX - COMPLETED**

| Agree-REX Domain | Interclass Correlation Coefficient | 95% Confidence Interval | Rating |
| --- | --- | --- | --- |
| 1: Clinical Applicability | 0.84 | 0.59 to 0.95 | Good |
| 2: Values and Preferences | 0.71 | 0.28-0.91 | Moderate |
| 3: Implementability | 0.17 | -1.10 to 0.75 | Poor |
| 4: Total | 0.71 | 0.28 to 0.91 | Moderate |

# Appendix B

## Final CPG and BPS Reviewed

1. AASLD-IDSA. Recommendations for Testing, Managing, and Treating Hepatitis C | HCV Guidance.

2. Abara W.E., Qaseem A., Schillie S., et al. Hepatitis B vaccination, screening, and linkage to care: Best practice advice from the American College of Physicians and the Centers for Disease Control and Prevention. Ann Intern Med. 2017;167(11):794-804.

3. ACOG Committee on Health Care for Underserved Women. ACOG Committee Opinion No. 525: Health care for lesbians and bisexual women. Obstet Gynecol. 2012;119(5):1077-1080.

4. ACON. AOD LGBTIQ Inclusive Guidelines for Treatment Providers. Sydney, NSW, Australia: ACON; 2019.

5. Ad Hoc Expert Working Group Centre for Communicable Diseases and Infection Control. Human Immunodeficiency Virus HIV Screening and Testing Guide. Ottawa, Ontario, Canada: Public Health Agency of Canada; 2012.

6. Adelson SL. Practice Parameter on Gay, Lesbian, or Bisexual Sexual Orientation, Gender Nonconformity, and Gender Discordance in Children and Adolescents. J Am Acad Child Adolesc Psychiatry. 2012;51(9):957-974.

7. Aldrich KM and Sabol VK. GAPNA Consensus Statement on Proficiencies for the APRN Gerontological Specialist: Proficiency Statement 6. Geriatr Nurs. 2016;37(5):412-414.

8. Alempijevic D, Beriashvili R, Beynon J, et al. Statement of the Independent Forensic Expert Group on Conversion Therapy. Torture. 2020;30(1):66-78.

9. Altarum Institute. Sexual Health and Your Patients: A Provider’s Guide. Washington, DC: Altarum Institute; 2022.

10. American Academy of Pediatrics Committee on Psychosocial Aspects of Child and Family Health. Promoting the well-being of children whose parents are gay or lesbian. Pediatrics. 2013;131(4):827-830.

11. American Geriatrics Society Ethics Committee. American Geriatrics Society care of lesbian, gay, bisexual, and transgender older adults position statement: American Geriatrics Society Ethics Committee. J Am Geriatr Soc. 2015;63(3):423-426.

12. Anonymous. Guidelines for HIV and AIDS student support services. J Sch Health. 1990;60(6):249-255.

13. Anonymous. Position statement: Homosexuality and the Immigration and Naturalization Service. Am J Psychiatry. 1991;148(11):1625.

14. Anonymous. Interventions to prevent HIV risk behaviors. NIH Consens Statement. 1997;15(2):1-41.

15. Anonymous. Guidelines: Prevention and Treatment of HIV and Other Sexually Transmitted Infections Among Men Who Have Sex with Men and Transgender People: Recommendations for a Public Health Approach 2011. Geneva, Switzerland: World Health Organization; 2011.

16. Anonymous. Health risks and needs of lesbian, gay, bisexual, transgender, and questioning adolescents position statement. J Pediatr Health Care. 2011;25(6):A9-10.

17. Anonymous. Consolidated Guidelines on HIV Prevention, Diagnosis, Treatment and Care for Key Populations - 2016 Update. Geneva, Switzerland: World Health Organization; 2016.

18. Anonymous. Recently acquired and early chronic hepatitis C in MSM: Recommendations from the European treatment network for HIV, hepatitis and global infectious diseases consensus panel. AIDS. 2020;34(12):1699-1711.

19. APA Commission on Psychotherapy by Psychiatrists. Position statement on therapies focused on attempts to change sexual orientation (reparative or conversion therapies). Am J Psychiatry. 2000;157(10):1719-1721.

20. Ard K and Makadon HJ. Improving the Health Care of Lesbian, Gay, Bisexual, and Transgender People: Understanding and Eliminating Health Disparities. Boston, MA: Fenway Health; 2016.

21. Australia LGBTAI Health. Current Evidence for Good Practice In Suicide Prevention For LGBTIQ+ People. Sydney, NSW, Australia: Australia LGBTAI Health; 2020.

22. Bartick M., Stehel E.K., Calhoun S.L., et al. Academy of breastfeeding medicine position statement and guideline: Infant feeding and lactation-related language and gender. Breastfeed Med. 2021;16(8):587-590.

23. Bekker L., Rebe K., Venter F., et al. Southern African guidelines on the safe use of pre-exposure prophylaxis in persons at risk of acquiring HIV-1 infection. S Afr Fam Pract (2004). 2016;58(5):6-17.

24. Bell S, Deen JF, Fuentes M, et al. Caring for American Indian and Alaska Native Children and Adolescents. Pediatrics. 2021;147(4):e2021050498.

25. Bhugra D., Gupta S., Bhui K., et al. WPA guidance on mental health and mental health care in migrants. World Psychiatry. 2011;10(1):2-10.

26. Bhugra D, Gupta S, Schouler-Ocak M, et al. EPA Guidance mental health care of migrants. Eur Psychiatry. 2014;29(2):107-115.

27. Bradley B, Kelts S, Robarge D, et al. NASN position statement: Sexual orientation and gender identity/expression (sexual minority students): school nurse practice. NASN Sch Nurse. 2013;28(2):112-113.

28. Brady M.T., Byington C.L., Davies H.D., et al. HPV vaccine recommendations. Pediatrics. 2012;129(3):602-605.

29. Bridges C.B. Recommended adult immunization schedule: United States, 2012. Ann Intern Med. 2012;156(3):211-217.

30. Broeckaert L. Practice Guidelines in Peer Health Navigation for People Living with HIV. Toronto, ON, Canada: Canadian AIDS Treatment Information Exchange; 2018.

31. Caceres BA, Streed CG, Corliss HL, et al. Assessing and Addressing Cardiovascular Health in LGBTQ Adults: A Scientific Statement from the American Heart Association. Circulation. 2020;142(19):e321-e332.

32. CANAC. Caring for Clients Who Are at Risk for or Living with HIV/AIDS. Canada: Canadian Association of Nurses in AIDS Care; 2013.

33. Center for Substance Abuse Treatment. Substance Abuse: Clinical Issues in Intensive Outpatient Treatment. Rockville, MD, USA: Substance Abuse and Mental Health Services Administration; 2006.

34. Centers for Disease Control and Prevention (CDC). US Public Health Service: Pre-Exposure Prophylaxis for the Prevention of HIV Infection in the United States, 2021 Update, A Clinical Practice Guideline. Atlanta, GA: Centers for Disease Control and Prevention; 2021:1-108.

35. Clutterbuck D, Asboe D, Barber T, et al. 2016 United Kingdom national guideline on the sexual health care of men who have sex with men. International journal of STD & AIDS. 2018;(a16, 9007917):956462417746897.

36. Committee On Adolescence. Office-based care for lesbian, gay, bisexual, transgender, and questioning youth. Pediatrics. 2013;132(1):198-203.

37. Committee on Adolescent Health Care. ACOG Committee Opinion No. 758: Promoting Healthy Relationships in Adolescents. Obstet Gynecol. 2018;132(5):e213-e220.

38. Committee on Gynecologic Practice and American Society for Reproductive Medicine. ACOG Committee Opinion No. 762: Prepregnancy Counseling. Obstet Gynecol. 2019;133(1):e78-e89.

39. Committee on Health Care for Underserved Women. ACOG Committee Opinion No. 749: Marriage and Family Building Equality for Lesbian, Gay, Bisexual, Transgender, Queer, Intersex, Asexual, and Gender Nonconforming Individuals. Obstet Gynecol. 2018;132(2):e82-e86.

40. Cordel H, Tantet C, Stempak T, et al. Addressing sexuality and sexual health with migrants. Practice guidelines. Infectious diseases now. 2022;52(2):61-67.

41. Crowcroft NS, Walsh B, Davison KL, et al. Guidelines for the control of hepatitis A virus infection. Communicable disease and public health. 2001;4(3):213-227.

42. Daniel H, Butkus R, and Health and Public Policy Committee of American College of Physicians. Lesbian, Gay, Bisexual, and Transgender Health Disparities: Executive Summary of a Policy Position Paper From the American College of Physicians. Ann Intern Med. 2015;163(2):135-137.

43. Davies S. Good Practice Guide: Many Shades of Blue: Enhancing Service Delivery to Address the Mental Health Needs of LGBTI Populations. Teneriffe, Queensland, Australia: Queensland AIDS Council; 2015:29.

44. Davis V. Lesbian Health Guidelines. Journal SOGC. 2000;22(3):202-205.

45. de Vries HJC, de Barbeyrac B, de Vrieze NHN, et al. 2019 European guideline on the management of lymphogranuloma venereum. Journal of the European Academy of Dermatology and Venereology : JEADV. 2019;33(10):1821-1828.

46. de Vries HJC, Nori AV, Kiellberg Larsen H, et al. 2021 European Guideline on the management of proctitis, proctocolitis and enteritis caused by sexually transmissible pathogens. Journal of the European Academy of Dermatology and Venereology : JEADV. 2021;35(7):1434-1443.

47. Earnshaw VA, Reisner SL, Juvonen J, et al. LGBTQ Bullying: Translating Research to Action in Pediatrics. Pediatrics. 2017;140(4):e20170432.

48. Emans SJ, Brown RT, Davis A, et al. Society for Adolescent Medicine Position Paper on Reproductive Health Care for Adolescents. The Journal of adolescent health : official publication of the Society for Adolescent Medicine. 1991;12(8):649-661.

49. Esser S, Schofer H, Hoffmann C, et al. S1 Guidelines for the Kaposi Sarcoma. Journal der Deutschen Dermatologischen Gesellschaft = Journal of the German Society of Dermatology : JDDG. 2022;20(6):892-904.

50. Ferri RL, Rosen-Carole CB, Jackson J, et al. ABM Clinical Protocol #33: Lactation Care for Lesbian, Gay, Bisexual, Transgender, Queer, Questioning, Plus Patients. Breastfeed Med. 2020;15(5):284-293.

51. Frankowski BL and American Academy of Pediatrics Committee on Adolescence. Sexual orientation and adolescents. Pediatrics. 2004;113(6):1827-1832.

52. Frasier Health Authority. Providing Diversity Competent Care to Gay Clients: A Handbook for Health Care Providers. Surrey, BC, Canada: Diversity Services, Fraser Health Authority; 2015.

53. Gay and Lesbian Medical Association. Guidelines for Care of Lesbian, Gay, Bisexual, and Transgender Patients. San Francisco CA: Gay and Lesbian Medical Association; 2006:1-35.

54. Gay and Lesbian Medical Association. Healthy People 2010: Companion Document for LGBT Health.; 2010.

55. Grasso C, McDowell MJ, Goldhammer H, et al. Planning and implementing sexual orientation and gender identity data collection in electronic health records. J Am Med Inform Assoc. 2019;26(1):66-70.

56. Griggs J, Maingi S, Blinder V, et al. American Society of Clinical Oncology Position Statement: Strategies for Reducing Cancer Health Disparities Among Sexual and Gender Minority Populations. J Clin Oncol. 2017;35(19):2203-2208.

57. Horner P.J., Blee K., Falk L., et al. 2016 European guideline on the management of non-gonococcal urethritis. International Journal of STD and AIDS. 2016;27(11):928-937.

58. Hughes TL, Jackman K, Dorsen C, et al. How can the nursing profession help reduce sexual and gender minority related health disparities: Recommendations from the National Nursing LGBTQ Health Summit. Nursing outlook. 2022;70(3):513-524.

59. Janier M, Unemo M, Dupin N, et al. 2020 European guideline on the management of syphilis. Journal of the European Academy of Dermatology and Venereology : JEADV. 2021;35(3):574-588.

60. Jespers V., Stordeur S., Berghe W.V., et al. Diagnosis and treatment of gonorrhea: 2019 Belgian National guideline for primary care. Acta Clinica Belgica: International Journal of Clinical and Laboratory Medicine. 2022;77(1):186-194.

61. Kohli M, Medland N, Fifer H, et al. BASHH updated position statement on doxycycline as prophylaxis for sexually transmitted infections. Sexually transmitted infections. 2022;98(3):235-236.

62. Lamont J, Bajzak K, Bouchard C, et al. No. 279-Female Sexual Health Consensus Clinical Guidelines. J Obstet Gynaecol Can. 2018;40(6):e451-e503.

63. Lanjouw E., Ouburg S., de Vries H.J., et al. 2015 European guideline on the management of Chlamydia trachomatis infections. International Journal of STD and AIDS. 2016;27(5):333-348.

64. Lavin A, LaMonte Askew G, Baum R, et al. Runaway youth: Caring for the nation’s largest segment of missing children. Pediatrics. 2020;145(2).

65. Martinez-Gomez X, Curran A, Campins M, et al. Multidisciplinary, evidence-based consensus guidelines for human papillomavirus (HPV) vaccination in high-risk populations, Spain, 2016. Euro surveillance : bulletin European sur les maladies transmissibles = European communicable disease bulletin. 2019;24(7).

66. Mast EE, Weinbaum CM, Fiore AE, et al. A comprehensive immunization strategy to eliminate transmission of hepatitis B virus infection in the United States: recommendations of the Advisory Committee on Immunization Practices (ACIP) Part II: immunization of adults. MMWR Recommendations and reports : Morbidity and mortality weekly report Recommendations and reports. 2006;55(RR-16):1-4.

67. Meites E, Szilagyi P, Chesson HW, et al. Human Papillomavirus Vaccination for Adults: Updated Recommendations of the Advisory Committee on Immunization Practices. MMWR Morb Mortal Wkly Rep. 2019;68(32):698-702.

68. Meyers D., Wolff T., Gregory K., et al. USPSTF recommendations for STI screening. American Family Physician. 2008;77(6):819-824.

69. Moyer V.A. and US Preventative Services Task Force. Screening for HIV: U.S. Preventive Services Task Force recommendation statement. Ann Intern Med. 2013;159(1):51-60.

70. National LGBTQIA+ Health Education Center. Emergency Preparedness and Lesbian, Gay, Bisexual & Transgender (LGBT) People: What Health Centers Need to Know. Boston, MA: The Fenway Institute; 2016.

71. National LGBTQIA+ Health Education Center. Providing Inclusive Services and Care for LGBT People: A Guide for Healthcare Staff. Boston, MA: The Fenway Institute; 2016.

72. National LGBTQIA+ Health Education Center. Providing Trauma-Informed Care at Health Centers for HIV-Positive Men Who Have Sex with Men. Boston, MA: The Fenway Institute; 2017.

73. National LGBTQIA+ Health Education Center. Addressing Eating Disorders, Body Dissatisfaction, and Obesity Among Sexual and Gender Minority Youth. Boston, MA: The Fenway Institute; 2018.

74. National LGBTQIA+ Health Education Center. Addressing Opioid Use Disorder among LGBTQ Populations. Boston, MA: The Fenway Institute; 2018.

75. National LGBTQIA+ Health Education Center. Addressing HIV and Sexually Transmitted Infections among LGBTQ People: A Primer for Health Centers. Boston, MA: The Fenway Institute; 2019.

76. National LGBTQIA+ Health Education Center. Diabetes Prevention and Management for LGBTQ People. Boston, MA: The Fenway Institute; 2019.

77. National LGBTQIA+ Health Education Center. Promoting the Behavioral Health of LGBT Older Adults. Boston, MA: The Fenway Institute; 2019.

78. National LGBTQIA+ Health Education Center. Recognizing and Addressing Intimate Partner Violence in Relationships of LGBTQ People: A Primer for Health Centers. Boston, MA: The Fenway Institute; 2019.

79. National LGBTQIA+ Health Education Center. 10 Strategies for Creating Inclusive Health Care Environments for LGBTQIA+ People. Boston, MA: The Fenway Institute; 2021:15.

80. National LGBTQIA+ Health Education Center. A Quick Guide for Supporting the Behavioral Health of Sexual Minority Men | 2021. Boston, MA: The Fenway Institute; 2021.

81. National LGBTQIA+ Health Education Center. Housing, Health, and LGBTQIA+ Older Adults. Boston, MA: The Fenway Institute; 2021.

82. National LGBTQIA+ Health Education Center. LGBTQIA+ Youth and Experiences of Human Trafficking: A Healing Centered Approach. Boston, MA: The Fenway Institute; 2021.

83. National LGBTQIA+ Health Education Center. Sexual Health Care for Older LGBTQIA+ Adults. Boston, MA: The Fenway Institute; 2021.

84. National LGBTQIA+ Health Education Center. Health Care Considerations for Two Spirit and LGBTQIA+ Indigenous Communities. Boston, MA: The Fenway Institute; 2022.

85. National LGBTQIA+ Health Education Center. READY, SET, GO!: A Guide for Collecting Data on Sexual Orientation and Gender Identity. Boston, MA: The Fenway Institute; 2022.

86. Palfreeman A., Sullivan A., Rayment M., et al. British HIV Association/British Association for Sexual Health and HIV/British Infection Association adult HIV testing guidelines 2020. HIV Med. 2020;21(S6):1-26.

87. Pan American Health Organization. Consolidated Recommendations For: Prevention, Care and Treatment of Viral Hepatitis B and C Coinfection in People Living with HIV. Geneva, Switzerland: World Health Organization; 2019:1-8.

88. Petrosky E, Bocchini JAJ, Hariri S, et al. Use of 9-valent human papillomavirus (HPV) vaccine: updated HPV vaccination recommendations of the advisory committee on immunization practices. MMWR Morbidity and mortality weekly report. 2015;64(11):300-304.

89. Pineda J.A., Climent B., Garcia F., et al. Executive summary: Consensus document of GEHEP of the Spanish Society of Infectious Diseases and Clinical Microbiology (SEIMC), along with SOCIDROGALCOHOL, SEPD and SOMAPA on hepatitis C virus infection management in drug users. Enfermedades Infecciosas y Microbiologia Clinica. 2020;38(3):127-131.

90. Poirier JM, Francis KB, Fisher SK, et al. Practice Brief 1: Providing Services and Supports for Youth who are Lesbian, Gay, Bisexual, Transgender, Questioning, Intersex or Two-Spirit. 2008.

91. Rainbow Health Ontario. LGBTQ Youth Suicide. Toronto, ON, Canada: Sherbourne Health; 2013.

92. Rainbow Health Ontario. LGBTQ People and Eating. Toronto, ON, Canada: Sherbourne Health; 2014.

93. Rainbow Health Ontario. LGBTQ People and Exercise. Toronto, ON, Canada: Sherbourne Health; 2015.

94. Rainbow Health Ontario. LGBTQ People, Drug Use & Harm Reduction. Toronto, ON, Canada: Sherbourne Health; 2015.

95. Rainbow Health Ontario. Intimate Partner Violence in LGBTQ Communities. Toronto, ON, Canada: Sherbourne Health; 2016.

96. Rainbow Health Ontario. LGBTQ Cancer Factsheet. Toronto, ON, Canada: Sherbourne Health; 2016.

97. Rainbow Health Ontario. Health Equity Impact Assessment: LGBT2SQ Populations Supplement. Toronto, ON, Canada: Sherbourne Health; 2017.

98. Rainbow Health Ontario. LGBT2SQ-Seniors: An Evidence Review and Practical Guide Designed for Healthcare Providers and Researchers. Toronto, ON, Canada: Sherbourne Health; 2021.

99. Rainbow Health Ontario. Health in Focus: 2SLGBTQ Sexual Health. Toronto, ON, Canada: Sherbourne Health; 2022:17.

100. Rainbow Health Ontario. Health in Focus: Racialized 2SLGBTQ Health. Toronto, ON, Canada: Sherbourne Health; 2022:18.

101. Randell A and Scanlan F. Headspace Evidence to Practice: A Guide for Clinicians Working Safely and Inclusively with Sexuality Diverse Young People. Sydney, NSW, Australia: Headspace; 2019.

102. Rockstroh J.K. Acute hepatitis C in HIV-infected individuals: Recommendations from the European AIDS Treatment Network (NEAT) consensus conference: The European AIDS Treatment Network (NEAT) Acute Hepatitis C Infection Consensus Panel. AIDS. 2011;25(4):399-409.

103. Rogstad K., Thomas A., Williams O., et al. UK national guideline on the management of sexually transmitted infections and related conditions in children and young people (2009). International Journal of STD and AIDS. 2010;21(4):229-241.

104. Ryan C. A Practitioner’s Resource Guide: Helping Families to Support Their LGBT Children. Rockville, MD, USA: Substance Abuse and Mental Health Services Administration; 2014:18.

105. Sauvageau C and Dufour-Turbis C. HPV vaccination for MSM: Synthesis of the evidence and recommendations from the Quebec Immunization Committee. Human vaccines & immunotherapeutics. 2016;12(6):1560-1565.

106. Savage J., Pell C., Pierce A., et al. National guidelines for post-exposure prophylaxis after non-occupational exposure to HIV. Sexual Health. 2007;4(4):277-283.

107. Sexually Transmissible Infections in Gay Men Action Group (STIGMA). Australian Sexually Transmitted Infection & HIV Testing Guidelines 2019 For Asymptomatic Men Who Have Sex with Men. New South Wales, Australia: New South Wales STI Programs Unit; 2019.

108. Sindhuja T, Gupta V, Bhari N, et al. Asian guidelines for genital herpes. Journal of Infection and Chemotherapy. 2021;27(10):1389-1399.

109. Society for Adolescent Health and Medicine. Recommendations for promoting the health and well-being of lesbian, gay, bisexual, and transgender adolescents: a position paper of the Society for Adolescent Health and Medicine. J Adolesc Health. 2013;52(4):506-510.

110. Steinke EE, Jaarsma T, Barnason SA, et al. Sexual counselling for individuals with cardiovascular disease and their partners: a consensus document from the American Heart Association and the ESC Council on Cardiovascular Nursing and Allied Professions (CCNAP). Eur Heart J. 2013;34(41):3217-3235.

111. Stewart DE, MacMillan H, and Wathen N. Intimate partner violence. Canadian journal of psychiatry Revue canadienne de psychiatrie. 2013;58(6):Insert-17.

112. Suprina JS, Matthews CH, Kakkar S, et al. Best Practices in Cross-Cultural Counseling: The Intersection of Spiritual/Religious Identity and Affectional/Sexual Identity. Journal of LGBT Issues in Counseling. 2019;13(4):293-325.

113. Tan DHS, Hull MW, Yoong D, et al. Canadian guideline on HIV pre-exposure prophylaxis and nonoccupational postexposure prophylaxis. CMAJ. 2017;189(47):E1448-E1458.

114. The American College of Obstetricians and Gynecologists. Marriage and Family Building Equality for Lesbian, Gay, Bisexual, Transgender, Queer, Intersex, Asexual, and Gender Nonconforming Individuals. 2018;132(2):5.

115. US Preventive Services Task Force, Krist AH, Davidson KW, et al. Screening for Hepatitis B Virus Infection in Adolescents and Adults: US Preventive Services Task Force Recommendation Statement. JAMA. 2020;324(23):2415-2422.

116. US Preventive Services Task Force, Owens DK, Davidson KW, et al. Preexposure Prophylaxis for the Prevention of HIV Infection: US Preventive Services Task Force Recommendation Statement. JAMA. 2019;321(22):2203.

117. Veltman A and Chaimowitz G. Mental Health Care for People Who Identify as Lesbian, Gay, Bisexual, Transgender, and (or) Queer. Can J Psychiatry. 2014;59(11):1-7.

118. White J., O’Farrell N., and Daniels D. 2013 UK National Guideline for the management of lymphogranuloma venereum: Clinical Effectiveness Group of the British Association for Sexual Health and HIV (CEG/BASHH) Guideline development group. Int J STD AIDS. 2013;24(8):593-601.

119. Workowski KA, Bachmann LH, Chan PA, et al. Sexually Transmitted Infections Treatment Guidelines, 2021. MMWR Recomm Rep. 2021;70(4):1-187.

120. Yen-Hao Chu I, Wen-Wei Ku S, Li C-W, et al. Taiwan guideline on oral pre-exposure prophylaxis for HIV prevention - 2018 update. J Microbiol Immunol Infec. 2020;53(1):1-10.

121. Zuniga JM, Bekker L-G, Montaner J, et al. IAPAC guidelines for optimizing the HIV care continuum for adults and adolescents. J Int Assoc Provid AIDS Care. 2015;14(Supplement 1):S3-S34.

# Appendix C

## Search Strategy

## Ovid MEDLINE(R) ALL <1946 to July 08, 2022>

## 1 exp clinical pathway/

## 2 exp clinical protocol/

## 3 exp consensus/

## 4 exp consensus development conference/

## 5 exp consensus development conferences as topic/

## 6 critical pathways/

## 7 exp guideline/

## 8 guidelines as topic/

## 9 exp practice guideline/

## 10 practice guidelines as topic/

## 11 health planning guidelines/

## 12 (guideline or practice guideline or consensus development conference or consensus development conference, NIH).pt.

## 13 (position statement* or policy statement* or practice parameter* or best practice*).ti,ab,kf,kw.

## 14 (standards or guideline or guidelines).ti,kf,kw.

## 15 ((practice or treatment* or clinical) adj guideline*).ab.

## 16 (CPG or CPGs).ti.

## 17 consensus*.ti,kf,kw.

## 18 consensus*.ab. /freq=2

## 19 ((critical or clinical or practice) adj2 (path or paths or pathway or pathways or protocol*)).ti,ab,kf,kw.

## 20 recommendat*.ti,kf,kw.

## 21 (care adj2 (standard or path or paths or pathway or pathways or map or maps or plan or plans)).ti,ab,kf,kw.

## 22 (algorithm* adj2 (screening or examination or test or tested or testing or assessment* or diagnosis or diagnoses or diagnosed or diagnosing)).ti,ab,kf,kw. 9459

## 23 (algorithm* adj2 (pharmacotherap* or chemotherap* or chemotreatment* or therap* or treatment* or intervention*)).ti,ab,kf,kw.

## 24 1 or 2 or 3 or 4 or 5 or 6 or 7 or 8 or 9 or 10 or 11 or 12 or 13 or 14 or 15 or 16 or 17 or 18 or 19 or 20 or 21 or 22 or 23

## 25 "sexual and gender minorities"/

## 26 sexual minor*.mp.

## 27 gender minor*.mp.

## 28 lesbian*.mp.

## 29 gay*.mp.

## 30 homosexual*.mp.

## 31 bisexual*.mp.

## 32 queer*.mp.

## 33 asexual*.mp.

## 34 pansexual*.mp.

## 35 two spirit*.mp.

## 36 GLBT*.mp.

## 37 LGB*.mp.

## 38 2slgb*.mp.

## 39 men who have sex with men.mp.

## 40 MSM.mp.

## 41 gbMSM.mp.

## 42 women who have sex with women.mp.

## 43 WSW.mp.

## 44 LBWSW.mp.

## 45 25 or 26or 27 or 28 or29 or 30 or 31 or 32 or 33 or 34 or 35 or 36 or 37 or 38 or 39 or 40 or 41 or 42 or 43 or 44

## 46 24 and 45

## 47 limit 46 to english language

## 48 limit 46 to animals

49 47 not 48

Box C: Inclusion and Exclusion Criteria

**Exclusion Criteria**

1. Review articles, including narrative reviews or general overviews of trans care that do not make systematic clinical recommendations or are focused on one clinical question.

2. Experimental or observational studies, editorials, or letters to the editor.

3. Of regional or single hospital system or community in scope (a provincial or state guideline that is applicable to those outside the state or nationally is acceptable, a local policy or guidance document meant for a local audience only is not).

4. Non-medical guidelines.

5. If a more recent version of the guidelines exists, then the earlier version will be excluded.

**Inclusion Criteria**

1. Any clinical practice guidelines, best practice statement, consensus document, critical pathway, or other systematic documents outlining holistic standard of care recommendations for transgender or gender non-binary/nonconforming persons.

2. Medical or para-medical in scope.

3. In any setting, community, or hospital.

4. In English.

5. Any region or nation.

6. Of national, or international in scope (see note in exclusion criteria).

##

## Extraction Template

Study ID:

Title:

Author and Year Published:

Country/Region in which document is relevant:

- United States
- United Kingdom
- Canada
- Australia
- North America
- Caribbean Islands
- South America
- Europe
- Africa
- Asia
- International
- Other: ____________

Source

- Peer-reviewed article
- Non-peer reviewed article

Does the document include recommendations for care in the Emergency Department?

- Yes
- No

If Yes to above, what stage of care does it concern, and what is/are the recommendation(s)?

- Decision to Come to ED: ____
- Prehospital Care: ____
- Registration: ____
- Triage: ____
- Waiting Room: ____
- Rooming/Initial Nursing Care: ____
- History and Physical exam: ____
- Investigations: ____
- Diagnoses : ____
- Treatment : ____
- Disposition/Discharge Planning: ____
- Follow-up care: ____

Does this meet the definition of a clinical practice guideline or one of a best practice statement?

- Clinical Practice Guideline
- Best Practice Statement
